# Human, animal, water source interactions and leptospirosis in Thailand

**DOI:** 10.1038/s41598-021-82290-5

**Published:** 2021-02-05

**Authors:** Udomsak Narkkul, Janjira Thaipadungpanit, Nattachai Srisawat, James W. Rudge, Metawee Thongdee, Rungrawee Pawarana, Wirichada Pan-ngum

**Affiliations:** 1 Department of Tropical Hygiene, Faculty of Tropical Medicine, Mahidol University, Bangkok, 10400 Thailand; 2 Mahidol-Oxford Tropical Medicine Research Unit, Faculty of Tropical Medicine, Mahidol University, Bangkok, 10400 Thailand; 3 Department of Clinical Tropical Medicine, Faculty of Tropical Medicine, Mahidol University, Bangkok, 10400 Thailand; 4 Excellence Center for Critical Care Nephrology, King Chulalongkorn Memorial Hospital, Division of Nephrology, Department of Medicine, Critical Care Nephrology research Unit, and Tropical Medicine Cluster, Chulalongkorn University, Bangkok, 10330 Thailand; 5 Academy of Science, Royal Society of Thailand, Bangkok, 10300 Thailand; 6 Communicable Diseases Policy Research Group (CDPRG), Department of Global Health and Development, London School of Hygiene and Tropical Medicine, London, UK; 7 The Monitoring and Surveillance Center for Zoonotic Diseases in Wildlife and Exotic Animals (MoZWE), Faculty of Veterinary Science, Mahidol University, Nakhon Pathom, 73170 Thailand; 8 Center of Excellence for Biomedical and Public Health Informatics (BIOPHICS), Faculty of Tropical Medicine, Mahidol University, Bangkok, 10400 Thailand

**Keywords:** Health care, Health occupations, Risk factors

## Abstract

In Thailand, leptospirosis is primarily associated with those who work in agricultural occupations. Leptospirosis control is hampered by a poor understanding of the complex interactions between humans, animal reservoirs, *Leptospira*, and the variable spatial environment in which these factors coexist. We aimed to address key knowledge gaps concerning leptospirosis disease dynamics and the human–animal–water-source interface in two high-risk areas in Thailand. We conducted a cross-sectional survey among 746 study participants in two high-risk areas for leptospirosis in Thailand: Sisaket (SSK) and Nakhon Si Thammarat (NST). Interactions among humans, animals and water sources were quantified and analyzed. The presence of different animal species and thus contact patterns were different in NST and SSK. The consumption of water from the shared sources between the two areas was different. Those whose occupations were related to animals or environmental water and those who consumed water from more than two sources were more likely to have been infected with leptospirosis, with adjusted odds ratios 4.31 (95% CI 1.17–15.83) and 10.74 (95% CI 2.28–50.53), respectively. Understanding specific water-source sharing networks and human–animal contact patterns is useful when designing national and area-specific control programmes to prevent and control leptospirosis outbreaks.

## Introduction

Leptospirosis is a zoonotic disease caused by a spirochete bacterium, *Leptospira*, and can affect humans and other mammals. With more than 1,000,000 cases and 50,000 deaths estimated to occur in humans each year, leptospirosis is one of the most prevalent zoonotic diseases worldwide^1–4^. Humans can acquire this infection via the exposure of a wound or mucous membrane to the bacteria. This exposure can occur through direct contact with animals, as well as via indirect contact following exposure to water or soil that has been contaminated by the urine of infected animals^2,5–9^. A variety of animals, including rats, horses, cows, dogs, cats, buffaloes and pigs, can carry pathogenic *Leptospira* in their kidneys during an infection and for weeks after becoming infected, during which time they excrete the pathogen in their urine, subsequently infecting other animals or humans^10–13^. Rodents are also chronic asymptomatic carriers of the pathogenic *Leptospira*^14^. Individual humans with leptospirosis can present a wide range of symptoms, from being asymptomatic to conditions that involve multiple organs^5,9,12^. Any delay in seeking treatment (considered to be beyond three days after onset) significantly increases the risk of death^15^.

In Thailand, the *Leptospira* species that most frequently infects the human population is *L*. *interrogans*, while the most predominant serovar is Autumnalis^16^. Leptospirosis was first reported in Thailand in 1942. The annual number of reported leptospirosis cases increased from 398 cases in 1996 to 14,285 cases in 2000. In 2001, 2002, and 2003, the number of reported cases decreased but remained high, at 10,217, 6864, and 4958 cases, respectively^17^. From 2003 to 2012, there were a total of 41,089 cases of leptospirosis reported to the Bureau of Epidemiology, Ministry of Public Health, Thailand, giving an average annual incidence rate of 6.6 per 100,000 population. There were 606 deaths, i.e. a case fatality rate of 1.5%. The majority (72.3%) of cases were in individuals who worked in the agricultural sector, with incidence rates highest in males, people aged 55–64 years, people living in the northeastern region, and during the wet season^15^. Recently, the occurrence of leptospirosis has shifted geographically, and since 2015 many more cases have been reported from the southern part of Thailand. The northeastern and southern regions of Thailand are significantly different in many ways, including in their ecology, housing, and people’s lifestyles. However, the incidence of leptospirosis is high in both parts of the country, comprising almost 85% of all case reports each year since 2010^18^.

Advanced molecular methods have been used to assess the presence of *Leptospira* in the environment^19^. Specific and rapid detection of pathogenic *Leptospira* from contaminated soil and water sites using a variety of environmental samples is now possible given the refinement and expansion of PCR methods^20–24^. As a result, numerous reports have documented the presence of *Leptospira*, detected by PCR, in both soil and water samples, from various sources and in different geographical areas^25–31^.

The transmission of leptospirosis involves three components i.e. humans, animals and the environment. In the present study, we explored this neglected tropical disease under the “One Health” concept, which recognizes that the health of people is intricately connected to the health of animals and our shared environment^32,33^. In the context of Thailand, the interactions among these three components are very closely linked. For example, many people keep several types of animal in their house, and people often share water sources that are used for both agricultural and general consumption. Contact with animals while working and in daily life can lead to the direct transmission of leptospirosis, via contact with the urine of infected animals. More attention by research studies/control programmes, however, has been indirect transmission through contact with water contaminated with *Leptospira* by animal reservoirs. A number of initiatives to study leptospirosis in animals have already been undertaken in Thailand^11,13,30,34,35^. A better understanding of the contact patterns that drive leptospirosis transmission might help to identify options for infection prevention measures. In this study, we aimed to investigate and compare human–animal–environment (water sources) contacts in two high-risk settings in Thailand. The data were then analyzed to identify those factors associated with the incidence of leptospirosis in humans, for the future design of strategies to prevent and control the disease at both regional and national levels.

## Results

A total of 746 participants across the two study areas were interviewed between January and April 2017. Of these, 381 participants lived in Hua Suea sub-district, Khukhan district, Sisaket province, and 365 participants lived in Na Luang Sen sub-district, Thung Song, Nakhon Si Thammarat province.

**Figure 1:**
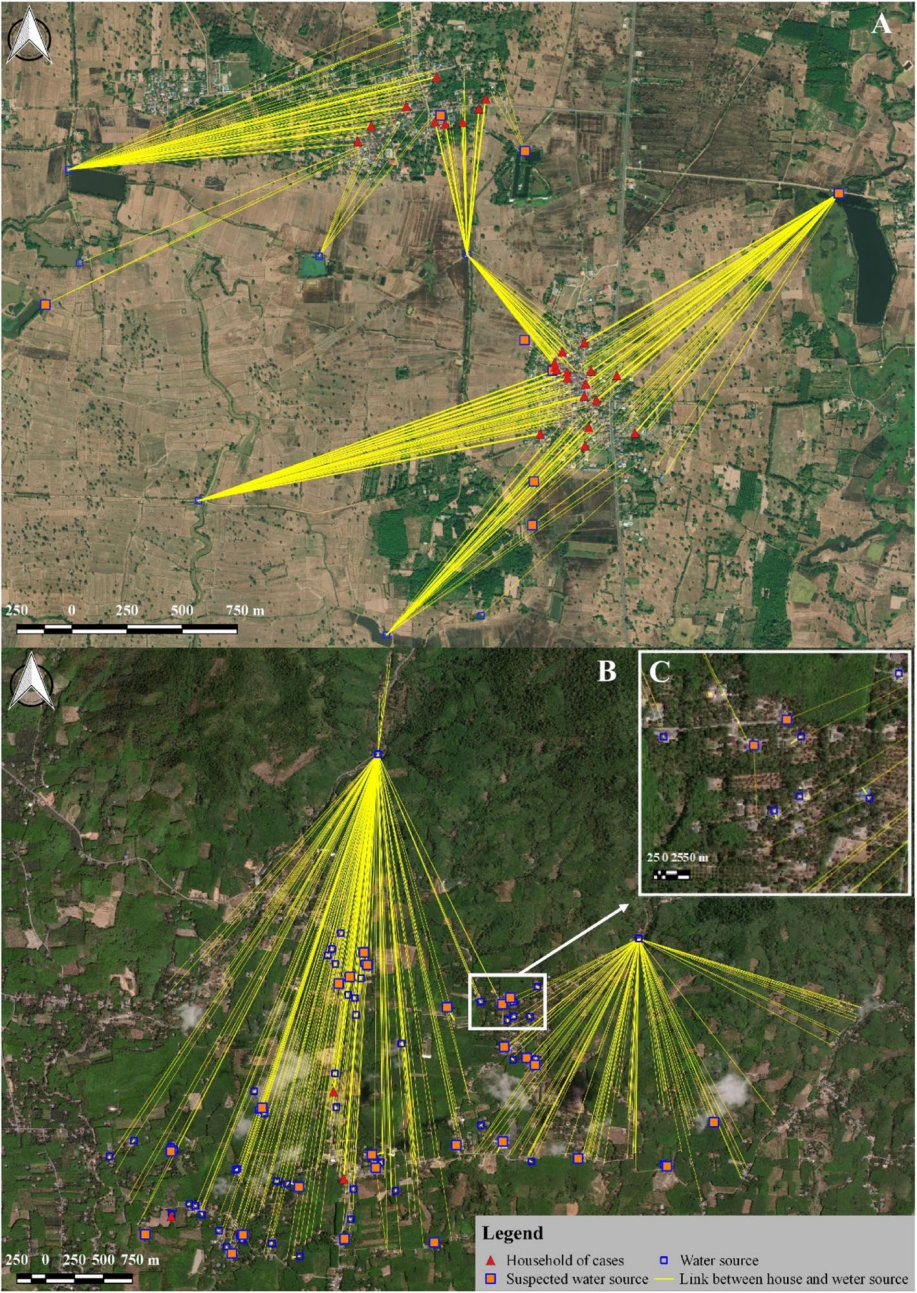
Surveyed households (n = 746) and water sources (n = 90) in (**A**) Hua Suea sub-district, Khukhan, Sisaket and (**B**) Na Luang Sen sub-district, Thung Song, Nakhon Si Thammarat. The location markers represent water sources. (**C**) A groundwater source owned by a household was usually close to or within the house. Quantum GIS version 3.12 (ESRI satellite) was used to map surveyed households and water sources (https://qgis.org/en/site/).

The average age of participants was 55 and 52 years in SSK and NST, respectively, and the majority of participants were female i.e. greater than 70% in both provinces shown in Table 1. The majority of those interviewed had attained a low level of education, and 100% of participants were of Thai ethnicity. A significant (*p* < 0.05) proportion of individuals in SSK were involved in animal- and/or environmental water-related work, i.e. rice/vegetable cultivation or animal farming, while the majority of individuals in NST were not involved in animal-related work, i.e. they worked on palm or rubber plantations. In general, the population in SSK had more contact with most types of animals, with the exception of domestic pets such as cats and dogs, whereas it was observed that most households in NST owned at least one dog. The level of protection worn when in contact with animals was significantly higher in NST (*p* < 0.05), and participants reported that they visited a private clinic whenever they felt unwell. These private clinics are mostly owned and running out of hours by public physicians. People who are better off would pay to go to private clinics when seeking care. In SSK, however, people were more likely to use the public sector when they sought treatment. With available universal health coverage scheme in the country, patients would pay very little (approximately one dollar) in a primary care service. Just 20% of participants living in SSK travelled outside of their own sub-district area at least once a week, compared with 80% of those living NST who travelled outside of their subdistrict at least once a week (Table 1). Figure 1 shows water-source sharing networks among households enrolled in this study. Overall, there were far fewer water sources available in SSK (n = 15) compared with the number of water sources in NST (n = 75). The main type of water source in SSK was a closed system for agricultural use, with an average of 21 households sharing each source. In SSK, most participants consumed water from more than one source, and often used at least one water source that was shared with others, whereas participants in NST mostly consumed water from just one source, usually a private groundwater source. The only common water sources shared among participants in NST were two waterfalls, which delivered water to local people via connected water pipes. Average number of households sharing the water from each waterfall was 183 (see Supplementary Fig. S1 online).

**Table 1:** Demographics and characteristics of study participants.

Demographic data	SSK (%) (n = 381)	NST (%) (n = 365)	Chi-square test	p-value
Age (${\overline{\text{x}}}$ ± SD) (t-test)	55.68 ± 12.61	52.55 ± 14.19	2.61	< 0.05
Sex				
Female	272 (71.4)	279 (76.4)	2.45	0.11
Male	109 (28.6)	86 (23.6)		
Education				
Primary school or lower	305 (80.05)	243 (66.58)	17.36	< 0.05
High school or above	76 (19.95)	122 (33.42)		
Ethnicity				
Thai	381 (100.0)	365 (100.0)	–	–
Occupation				
Unrelated to animal and/or environmental water	46 (12.07)	363 (99.45)	574.66	< 0.05
Related to animals and/or environmental water	335 (87.93)	2 (0.55)		
Travels outside of their sub-district at least once a week				
No	309 (81.1)	71 (19.5)	283.50	< 0.05
Yes	72 (18.9)	294 (80.5)		
Contact with animals				
No	185 (48.56)	244 (61.37)	12.35	< 0.05
Yes	196 (51.44)	141 (38.63)		
Contact with cattle				
No	279 (73.23)	345 (94.52)	61.78	< 0.05
Yes	102 (26.77)	20 (5.48)		
Contact with farm animals				
No	235 (61.68)	282 (77.26)	21.27	< 0.05
Yes	146 (38.32)	83 (22.74)		
Contact with domestic pets				
No	305 (80.05)	249 (68.22)	13.65	< 0.05
Yes	76 (19.95)	116 (31.78)		
Protection (including wearing boots, avoiding contact with animals and showering after work)				
No	245 (64.30)	72 (19.73)	151.59	< 0.05
Yes	136 (35.70)	293 (80.27)		
Water source used				
One source	90 (23.62)	280 (76.71)	213.42	< 0.05
Two sources	258 (67.72)	82 (22.47)		
More than two sources	33 (8.66)	3 (0.82)

### Animal–environmental water source interactions

There were 90 water sources in the two study areas; 15 water sources were located in Hua Suea sub-district, Khukhan district, Sisaket province, and 75 water sources were located in Na Luang Sen sub-district, Thung Song, Nakhon Si Thammarat province. The water sources included environmental water surrounding households, ponds, groundwater, tap water, rivers/canals, and other standing water sources that could be accessed by humans, rodents, cattle (including cows and buffaloes), other farm animals (including chickens, ducks and pigs), and domestic animals (including cats and dogs). Further methodological details on the detection of *Leptospira* in the water samples collected in this survey and the risk associated with *Leptospira* contamination of the water sources can be found in Narkkul^36^.

Presence of animals around the water source, defined by direct observation of animal or their habitat, or confirmation of the participants, were found around 14 out of 15 water sources (93.3%) in SSK, while in NST animals were only found around 31 out of 75 water sources (41.3%). In SSK, the most common species found around water sources, in descending order, were buffaloes, cows, rodents, dogs, chickens, pigs and cats. For NST, the most common species found around water sources, in descending order, were dogs, chickens, cows, pigs, cats and ducks. Most water sources in SSK were used for agriculture (including livestock farming and horticulture), followed by sources for consumption (general water used apart from drinking), while most types of water sources in NST were consumption sources, followed by drinking sources and agricultural sources. Most water sources in both settings were closed systems, such as ponds and groundwater (Table 2).

**Table 2:** Characteristics of water sources in the study areas.

Characteristics	SSK (%) (n = 15)	NST (%) (n = 75)	Chi-square test	p-value
Animals around water sources				
No	1 (6.7)	44 (58.7)	13.22	< 0.05
Yes	14 (93.3)	31 (41.3)		
Rodents				
No	5 (33.3)	75 (100.0)	56.25	< 0.05
Yes	10 (66.7)	0		
Chickens				
No	12 (80.0)	55 (73.3)	0.29	0.58
Yes	3 (20.0)	20 (26.7)		
Cows				
No	2 (13.3)	72 (96.0)	58.43	< 0.05
Yes	13 (86.7)	3 (4.0)		
Buffaloes				
No	1 (6.7)	75 (100.0)	82.89	< 0.05
Yes	14 (93.3)	0		
Pigs				
No	14 (93.3)	73 (97.3)	0.62	0.43
Yes	1 (6.7)	2 (2.7)		
Dogs				
No	11 (73.3)	58 (77.3)	0.11	0.73
Yes	4 (26.7)	17 (22.7)		
Cats				
No	14 (93.3)	74 (98.7)	1.63	0.20
Yes	1 (6.7)	1 (1.3)		
Ducks				
No	15 (100.0)	74 (98.7)	0.20	0.65
Yes	–	1 (1.3)		
Drinking source				
No	15 (100.0)	30 (40.0)	18.00	< 0.05
Yes	–	45 (60.0)		
Consumption source				
No	13 (86.7)	24 (32.0)	15.42	< 0.05
Yes	2 (13.3)	51 (68.0)		
Agricultural source				
No	–	58 (77.3)	32.62	< 0.05
Yes	15 (100.0)	17 (22.67)		
Type of water source				
Closed system	13 (86.7)	72 (96.0)	2.07	0.15
Open system	2 (13.3)	3 (4.0)		

### Human–animal–environmental contact patterns

#### Human–animal contact patterns

**Figure 2:**
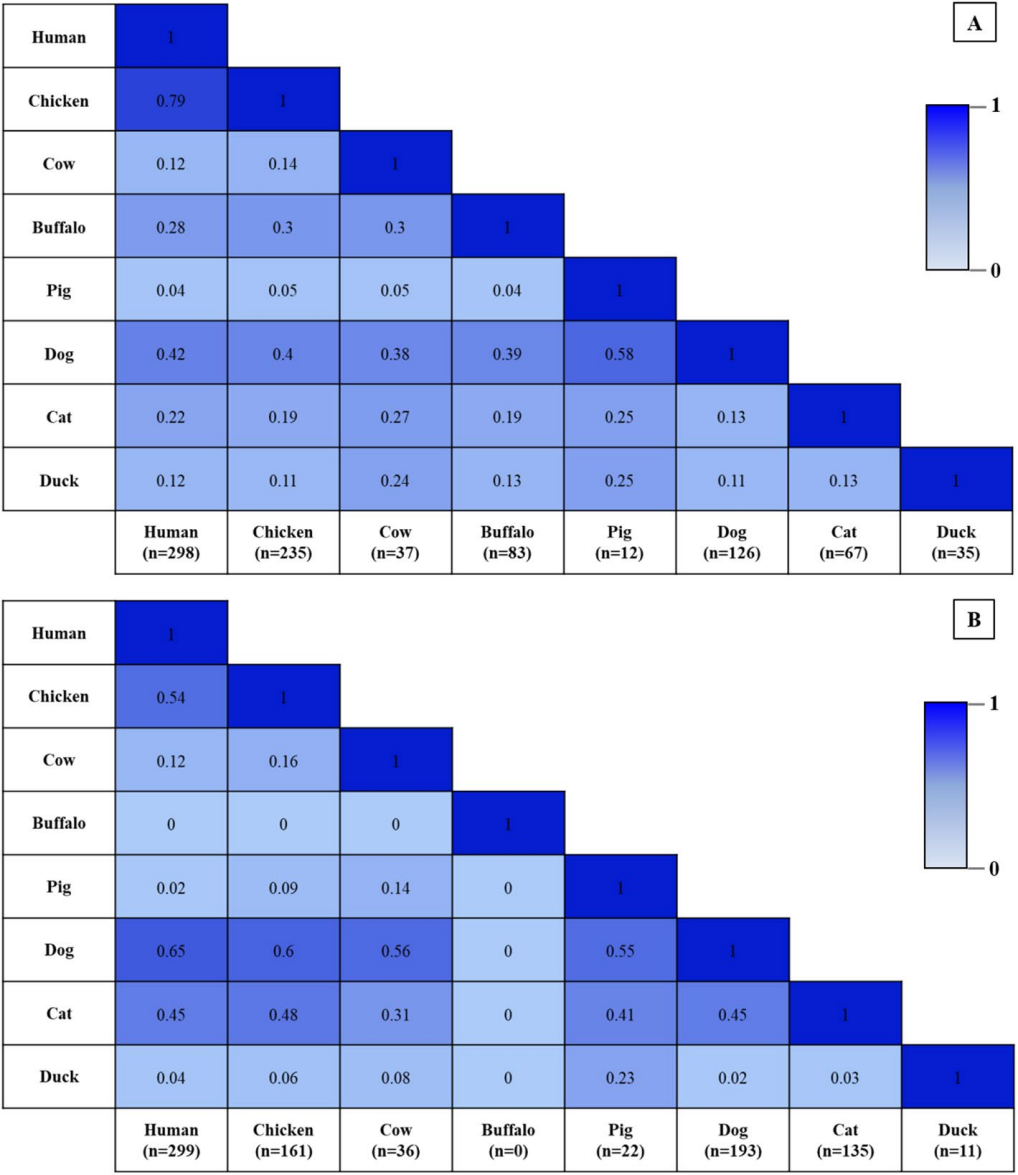
Coexistence between humans and different animal species in households surveyed, (**A**) SSK and (**B**) NST. The values in the cells show the proportion of households with different animal contacts.

The proportion of contacts between humans and animals, and between animals with other animals, was different in each setting. In SSK, the highest proportion of contacts was between humans with chickens, followed by humans with dogs, buffaloes, cats, cows, ducks and pigs, respectively. In NST, the proportion of contacts was highest between humans and dogs, followed by humans with chickens, cats, cows, ducks and pigs, respectively (Fig. 2).

#### Animal–environmental contact patterns

**Figure 3:**
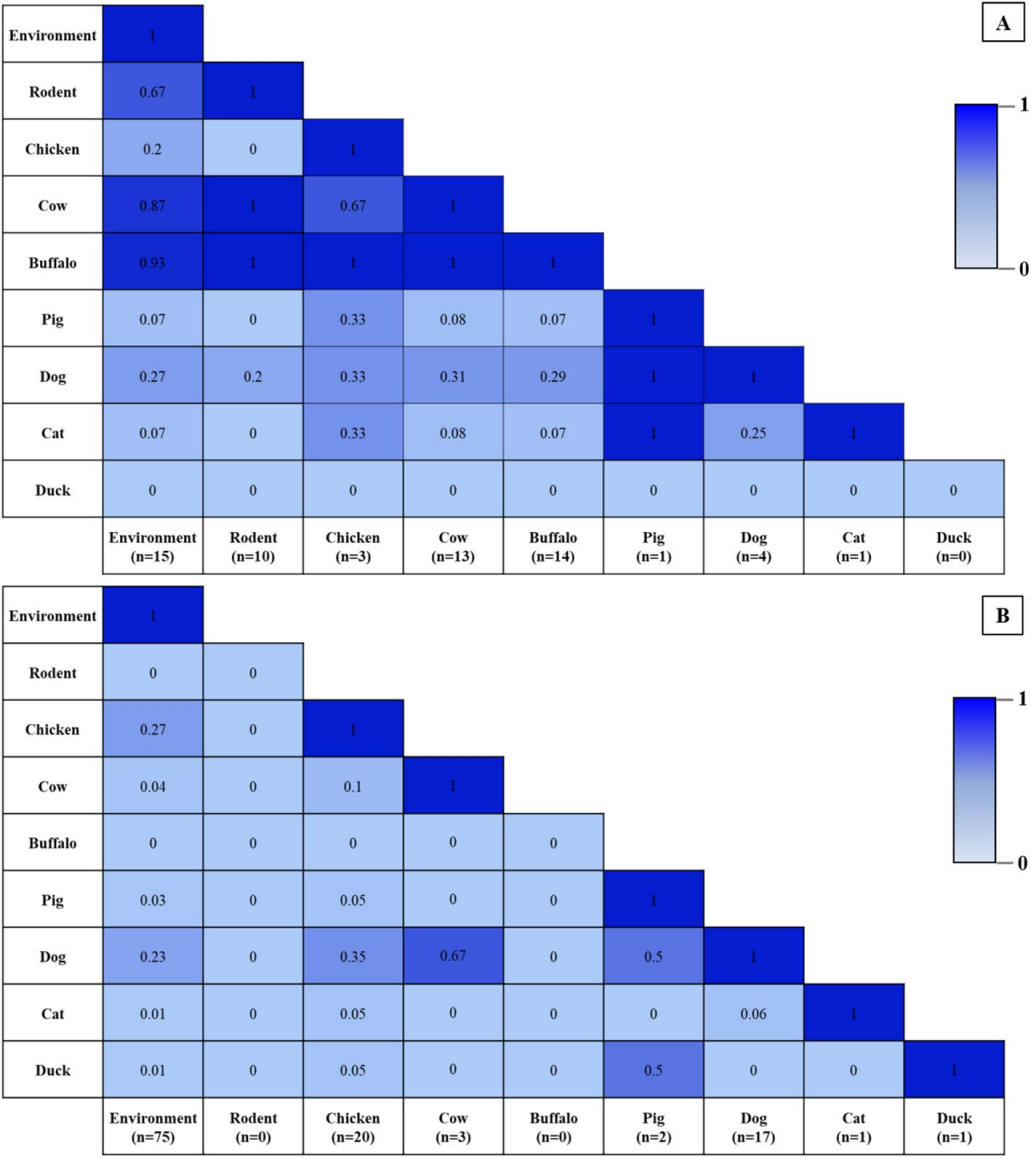
Coexistence between water sources and different animal species around water sources surveyed, (**A**) SSK and (**B**) NST. The values in the cells show the proportion of water sources with different animal contacts.

The proportion of contacts among different animal species with the water sources, and between animal species around the water sources, was different in each setting. In SSK, the highest proportion of contacts was between the water sources and buffaloes, followed by cows, rodents, dogs, chickens, cats and pigs, respectively. In NST, the proportion of contacts was highest between the water source and chickens, followed by dogs, cows, pigs, cats and ducks, respectively (Fig. 3).

### Risks associated with human leptospirosis

The findings from the regression analysis to investigate associations between leptospirosis and potential risk factors are summarized in Table 3. In SSK, age and the consumption of water from more than two sources were significantly associated with the risk of contracting leptospirosis (adjusted odds ratios (AOR) 0.96, 95% confidence intervals (CI), 0.92–0.99 and AOR 10.81, 95% CI 1.80–64.90, respectively), while for NST, no variables showed a significant association with the risk of contracting leptospirosis. All results discussed henceforth relate to those from the analysis where we combined the two study sites.

Three factors were found to be associated with leptospirosis: age, an occupation related to animals and/or environmental water, and consumption of water from more than two sources. Older people were less likely to be identified as having had leptospirosis (AOR 0.96, 95% CI 0.92–0.99). The age range covered in the study included individuals aged from 18 to more than 80 years. Individuals whose occupations were related to animal or environmental water were 4.31-times (95% CI 1.17–15.83) more likely to have been identified as a leptospirosis case, and those consuming water from more than two sources were 10.74-times (95% CI 2.28–50.53) more likely to have been identified as a leptospirosis case when compared with the results of participants who consumed water from one source (Table 3). It should be noted that, although the overall cases comprised more than 80% males, after matching these data to our survey (where more than 75% of participants were female), the sex ratio changed among the cases subjected to the analysis. Gender bias from sampling could also influence some behaviors associated with leptospirosis i.e. water consumption and contact with animals. At least in our study we found no significant differences in these behaviors between male and female participants. Caution should, however, be used in the interpretation of the risk factors.

**Table 3:** Univariate and multivariate mixed-effects logistic regression analysis for both settings.

Characteristics	n	Leptospirosis reports	OR (95% CI)	AOR (95% CI)
Age (${\overline{\text{x}}}$ ± SD)	54.15 ± 13.49		0.98 (0.95–1.01)	0.96 (0.92–0.99)
Sex				
Female	551 (73.86)	20 (3.63)	Ref	Ref
Male	195 (26.14)	8 (4.10)	1.10 (0.47–2.59)	1.17 (0.48–2.86)
Education level				
Primary school or lower	548 (73.46)	23 (4.20)	Ref	Ref
High school or higher	198 (26.54)	5 (2.53)	0.65 (0.24–1.79)	0.53 (0.17–1.68)
Occupation				
Unrelated to animal or environmental water	409 (54.83)	4 (0.98)	Ref	Ref
Related to animal or environmental water	337 (45.17)	24 (7.12)	7.76 (2.67–22.60)	4.31 (1.17–15.83)
Contact with animals				
No	409 (54.83)	15 (3.67)	Ref	Ref
Yes	337 (45.17)	13 (3.86)	0.89 (0.40–1.94)	1.07 (0.20–5.72)
Cattle				
No	624 (83.65)	22 (3.53)	Ref	Ref
Yes	122 (16.35)	6 (4.92)	1.08 (0.41–2.84)	0.58 (0.16–2.11)
Farm animals				
No	517 (69.30)	19 (3.68)	Ref	Ref
Yes	229 (30.70)	9 (3.93)	0.83 (0.36–1.95)	0.68 (0.18–2.49)
Domestic pets				
No	554 (74.26)	22 (3.97)	Ref	Ref
Yes	192 (25.74)	6 (3.12)	0.84 (0.33––2.17)	1.28 (0.34–4.75)
Travel outside sub-district				
No	81 (10.86)	4 (4.94)	Ref	Ref
Yes	665 (89.14)	24 (3.61)	1.12 (0.35–3.59)	0.91 (0.27–3.07)
Protection				
No	317 (42.49)	17 (5.36)	Ref	Ref
Yes	429 (57.51)	11 (2.56)	0.64 (0.27–1.51)	0.83 (0.34–2.05)
Water used				
One source	370 (49.60)	4 (1.08)	Ref	Ref
Two sources	340 (45.58)	18 (5.29)	4.80 (1.50–15.34)	2.64 (0.74–9.38)
More than two sources	36 (4.83)	6 (16.67)	17.02 (4.21–68.80)	10.74 (2.28–50.53)

## Discussion

This study demonstrated the importance of human demographics and behaviour, and interactions between humans, animals and the environment (specifically water sources), for the transmission of leptospirosis, an important zoonosis. Although people living in the study areas investigated here have a high risk of contracting leptospirosis, the areas differed significantly in terms of hosts and environmental factors. Northeast Thailand, where the highest number of leptospirosis cases was reported, is a high, flat plain, with relatively low humidity. Households often share several environmental water sources among them for use in agricultural work. A large proportion of the study population in this region have occupations that involve frequent exposure to animals and/or environmental bodies of water, such as rice paddy fields. The majority of animals found in this area are cattle and other farm animals. The occurrence of human leptospirosis is associated with agricultural work, as previously reported^37^. Strong links between cattle and human leptospirosis have also been revealed in previous work^11^. By comparison, studies of human leptospirosis in the south of Thailand are rare, but cases of infection have been reported in individuals who participated in white-water rafting^38^, as well as cases during flooding^39^. In southern Thailand, the landscape comprises mostly tropical rainforest, and the region is characterized by high humidity and rainfall. Most people work on rubber or palm plantations, rather than in livestock farming or other animal-related occupations. The majority of animals that participants reported owning or having contact with were domestic pets (in particular dogs) and farm animals such as chickens and pigs. Contact with cattle was rarely reported.

From a sociological prospective, people in the northeast of Thailand had a relatively lower level of education and took fewer protective measures against infectious diseases compared with measures taken by people living in southern Thailand (Table 1). In terms of household water consumption patterns, these two areas exhibited significant differences in sources of water for consumption and sharing patterns. In northeast Thailand, people tend to share water sources, which are relatively close to rice farming or animal husbandry areas. In southern Thailand, people tend to have their own water source, usually a groundwater source close to or within their house; common water sources shared among those living in the same area were waterfalls (Fig. 1).

Water-source sharing patterns can influence the risk of disease exposure. People who consume water from several sources, may face more chance to get leptospirosis infection if the water sources were contaminated with *Leptospira*. This is consistent with many previous studies, which showed that a high frequency of exposure to soil or water is a key risk factor for human leptospirosis^10,40,41^. Different water-source sharing patterns can also affect disease control strategies. For example, given a contaminated water source, it would be more challenging to contain an outbreak if it were in a northeastern province, where people are likely to consume water from the same source, than in a southern province where many households consume water from an individual groundwater source. This is in contrast with urban slum settings, where the water-related environmental risk of leptospirosis was being resident in flood-risk regions with open sewers^42^.

Occupations related to animals and environmental water, and the consumption of water from more than two sources, were shown to be statistically significant risk factors for human leptospirosis. Previous studies have shown that activities associated with agricultural and outdoor occupations are typically associated with *Leptospira* transmission and infection^40,41,43–46^. Older age groups showed a reduced risk of acquiring the infection. Several studies have shown that the highest leptospirosis risk occurs among young adults, aged between 15 and 34 years^3,47^. The lower risk of infection among older age groups may be explained by several factors, including: (1) protection by antibodies against *Leptospira* from previous exposures, which have been shown to confer immunity for a relatively short period of time^48^; (2) due to the matching of cases with the survey population which has a wide range of ages (from 18 to more than 80 years); and finally (3) the exposure to environmental water or animals among retired people is likely to be low, which might also explain the low risk of leptospirosis in this group. Finally, our study showed that consuming water from more than two sources was a strong risk factor for leptospirosis. This is consistent with our findings about the number of retrospective case reports versus the water-source sharing patterns in the two regions. Similar findings have been reported for many previous studies, showing that a high frequency of exposure to soil or water is a key risk factor for human leptospirosis^10,40,41,49,50^.

In terms of animal reservoirs, rodents have been identified as a key reservoir and the main focus for control of leptospirosis. The presence of rodents was also identified as the major risk for acquiring *Leptospira* antibodies in other settings, such as urban slums^42^. Recently, other types of animals have also been identified as playing a potentially important role in the transmission of leptospirosis to humans^11,30,37,51^. In agreement with previous studies that have investigated dogs in other parts of Thailand^30,52^, interacting with animals unrelated to work, such as dogs, is a potentially significant contributor to the transmission of leptospirosis, especially in the south of Thailand.

Our study had some limitations. First, the retrospective case reports which represented the disease burden in our risk association analysis may not have accurately represented the leptospirosis situation at the time when the survey was performed. Second, routine surveillance data often underestimate the true burden of disease, especially given that leptospirosis patients often present symptoms similarly to other febrile illnesses. Third, by matching the retrospective case reports to the household survey data, we were unable to consider all cases. All limitations on the use of case data could result in lowering the statistical power of the analyses. Finally, although there may have been many eligible persons in a household, we chose to select one person who was exposed to animals or environmental water sources in their daily activities, in order to study human–animal contact patterns. Thus, we are aware that the survey data might not fully represent the population in the study areas. In this study, we employed a novel way to quantify the interactions among humans, animals and environmental water sources, by observing the presence of animals around households and water sources and surveying household water consumption from different water sources. We further explored the quantity of these species in this context but decided to drop this idea as we considered that quantity may not be a good representative of the intensity of contact.

## Conclusion

In this study, we took a One Health approach to explore all three components, associated with the transmission of leptospirosis, i.e. humans, animals and environmental water sources, then quantify the interactions among them to identify factors associated with the risk of contracting leptospirosis. The contact patterns among different living hosts and living hosts with water sources varied geographically. In addition, the sharing of water sources was determined to be a risk factor for disease exposure. Although leptospirosis was found to be an occupational risk in the Thai context, the routine recommendations made by the health sector to self-protect, such as wearing boots or cleaning oneself after working in the field, may not be sufficient to control the disease. Knowledge relating to area-specific human–animal–water-source contact patterns and water-source sharing patterns should be taken into consideration when investigating leptospirosis outbreaks and planning strategies for outbreak prevention and control and, ultimately, the elimination of leptospirosis.

## Methods

### Study sites and population

**Figure 4:**
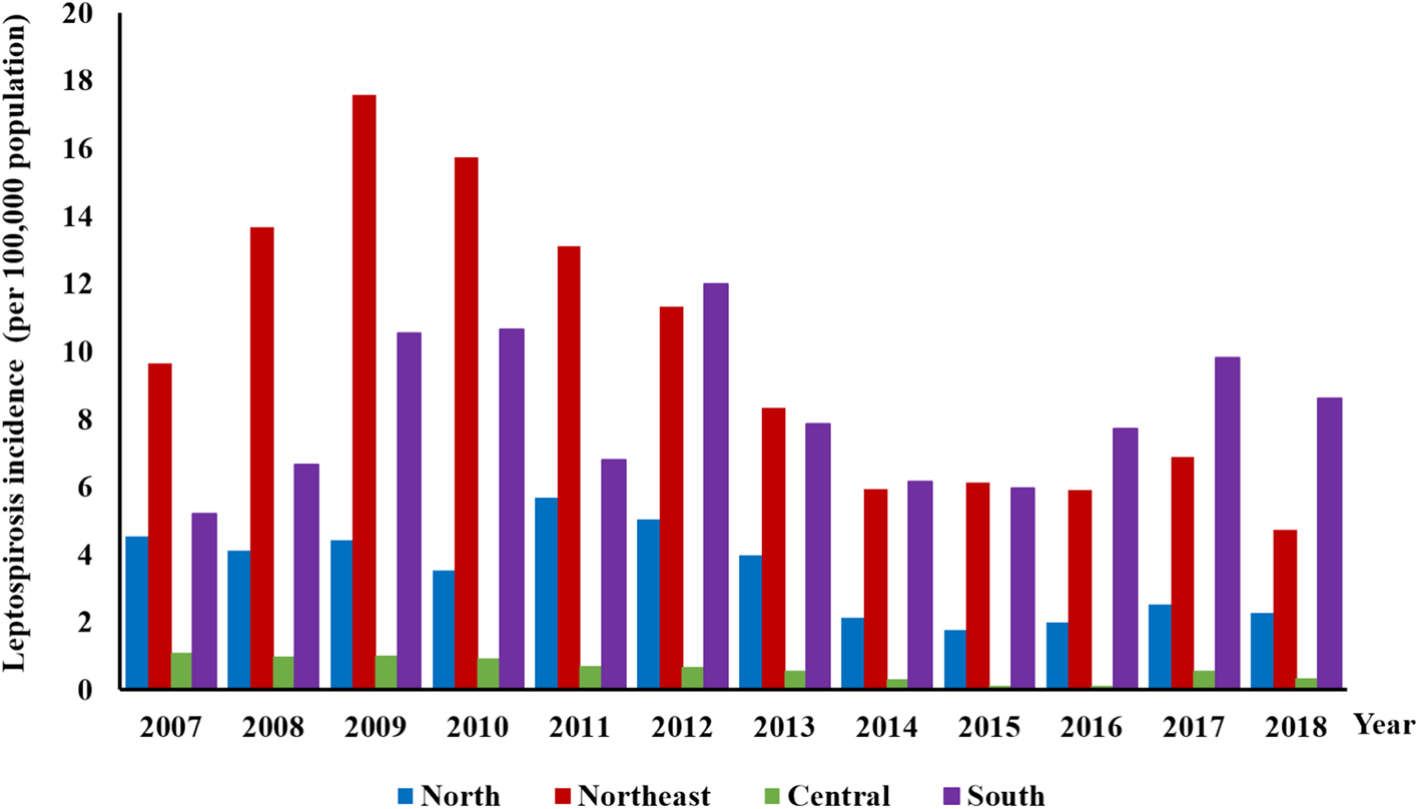
Distribution of human leptospirosis incidence by region in Thailand, 2007–2018.

This was a cross-sectional study, which recorded both individual- and household-level characteristics. Two provinces in Thailand were included in the study: Sisaket (SSK) and Nakhon Si Thammarat (NST). SSK is made up predominately of high, flat plains and its climate has relatively low humidity. Households often share several environmental water sources among them, for agricultural work. The landscape of NST comprises mostly tropical rainforest and has a climate with high humidity and rainfall. Household groundwater is a common source of water for human consumption. The populations in SSK and NST are 1,473,011 and 1,560,433, respectively. These provinces were selected based on their relatively high leptospirosis morbidity rates, as reported in national disease surveillance data and previous studies (Fig. 4^53^). In 2016, the morbidity rates of human leptospirosis in SSK and NST were 25.36/100,000 and 12.45/100,000 population, respectively, compared with a nationwide morbidity rate of 3.51/100,000 population^18^. The sub-districts with the highest morbidity were selected as the study sites: Khukhan district, SSK and Thung Song district, NST.

**Figure 5:**
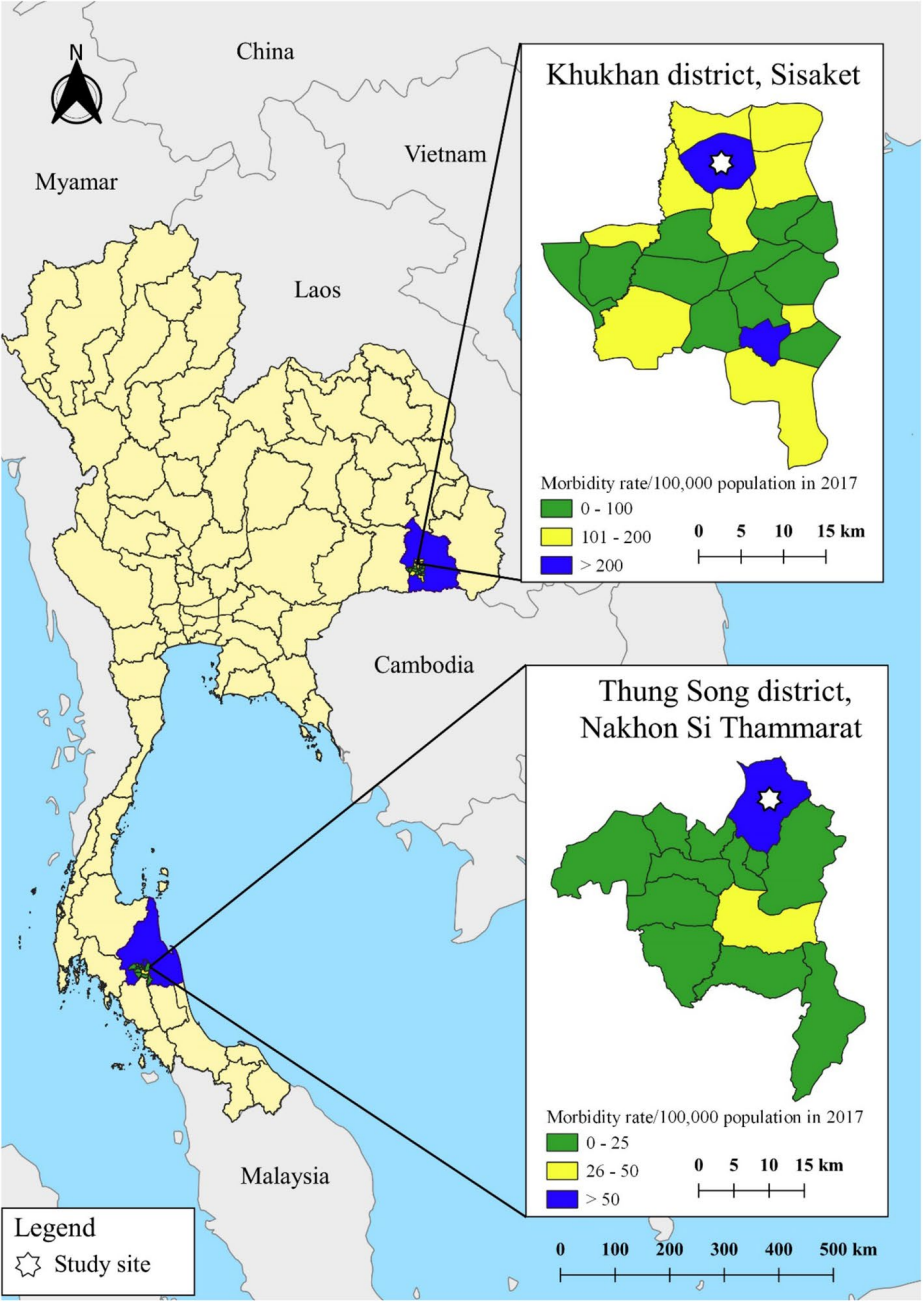
A map showing the study areas. Quantum GIS version 3.12 (ESRI basemaps) was used to generate the map (https://qgis.org/en/site/).

Between January and April 2017, a total of 746 households across the two study sites were identified and enrolled in the study. We selected villages in the two districts based on the number of case records held at the District Public Health Offices and the feasibility of performing the survey, based on the advice of sub-district health-promoting hospitals and village health volunteers. Households were selected based on their volunteering and signing an informed consent form to participate in the research. The sample sizes of 381 and 365 households in SSK and NST, respectively, were guided using *n4Studies* application^54^, by assuming the proportion of agricultural workers in SSK and NST to be 0.43 and 0.37, respectively^55^, with a precision of 0.05 and confidence level of 95%. The study population included five villages in Hua Suea sub-district, Khukhan, SSK (n = 381) and seven villages in Na Luang Sen sub-district, Thung Song, NST (n = 365), as shown in Fig. 5. These semi-rural villages were clustered in areas where there was a high incidence of leptospirosis reports. The number of households studied accounted for 50% and 23% in the sub-districts in SSK and NST, respectively.

### Data collection

A questionnaire designed to collect data relating to human contact patterns with animals and potential environmental sources was adapted from a previous study carried out in Cambodia (James W. Rudge, personal communications); the questionnaire was revised, in a paper form, following several rounds of discussion among the research team members. The questionnaire was divided into five main sections: (1) demographics: age, sex, occupation etc.; (2) travel patterns: frequency of travel outside the sub-district; (3) measures of human–animal contacts: types of animals, numbers of animals; (4) treatment-seeking behaviour: health-seeking behaviour, type of protection used to prevent disease, and suspected cause of fever; and (5) water sources and animal habitats: list of animals found around the water source(s), animals living around the water source(s) and type of water use, which were from direct observations of animals/their habitat made by the interviewer or by interviewing the participants (see Supplementary Questionnaire online). Staff at the Center of Excellence for Biomedical and Public Health Informatics (BIOPHICS) then developed the final version into a mobile (Android) phone application, which was subsequently used for data collection in this study. The software was tested in the field for its functionality, flow of questions and ease of exporting data to a database. Data extracted from the database were checked for accuracy and consistency.

The questionnaire was administered to one resident, aged at least 18 years, within each study household. Where more than one person was eligible to be interviewed per household, the person with the most potential contacts with animals and the environment, based on their lifestyle, was selected for the study. Global Positioning System (GPS) coordinates of the household and water sources used, as reported by the interviewee, were mapped and recorded using the mobile phone application. Maps were constructed using QGIS 3.12 to show the locations of the study areas (Fig. 5) and surveyed households and water sources (Fig. 1).

In addition, we obtained leptospirosis case reports between 2013 and 2017 from the relevant district health offices. During this period, a total of 93 and 10 cases of leptospirosis in humans in SSK and NST, respectively, were confirmed by immunofluorescence assay (IFA), the standard diagnostic test. The leptospirosis morbidity rate per 100,000 population in these two study areas was higher than the national level (average incidence of 27.71 and 12.12 in SSK and NST, respectively). Most cases occurred in people aged between 36 and 60 years, and 82.0% were in males. Leptospirosis cases were then matched to the addresses of survey participants. We identified 25 and 3 cases by address matching in SSK and NST, respectively, over the 5-year period. Among these cases, there were 8 males and 20 females, with an average age of 52.18 years.

Ethical approval for this study was obtained from the Ethics Committee of the Faculty of Tropical Medicine, Mahidol University, Bangkok, Thailand. The Certificate of Ethical Approval number is MUTM 2017-070-01. Informed consent was obtained from all participants and/or their legal guardians. All the methods were performed in accordance with relevant guidelines and regulations.

### Human–animal–environmental contact patterns

Observational data from the survey of the presence of animals around the households and water sources were used to quantify contact patterns in both study areas. Although these contacts were not directly measured in our study, it was assumed that the contacts were proportional to the coexistence between Class A—abiotic classes (i.e. households and water sources) and Class B—biotic classes (i.e. human and animals) with (see Table 2 and Supplementary Table S1 online for details), as follows:

$${\text{Intensity\,of\,contacts\,of\,class\,A\,to\,class\,B}} \propto \frac{No.\,Class\,A\,with\,the\,existence\,of\,Class\,B}{{No.\,Class\,A}}$$

### Statistical analysis

The survey data were analyzed for both descriptive and inferential statistics. Continuous variables, including age, were described using the mean and standard deviation. Independent categorical variables, including demographic data, travel patterns, human–animal contact information, treatment-seeking behaviour, water sources and animal habitats, were described using frequency, expressed as percentages. For the continuous variables, a *t*-test (two groups) was used to compare the groups. The chi-square test was used to examine bivariate associations between independent variables and the two study settings. A *p* value < 0.05 was considered statistically significant for the group comparison. To quantify the contact patterns among humans, animals and the environment, we analyzed the coexistence of two species, i.e. human vs animals and animals vs environmental water sources, based on the survey. To determine the risk of leptospirosis exposure, we mapped the survey data with the retrospective case report data at the sub-district level. Individual case records from the District Health Office, between 2013 and 2017, were mapped to their home address, followed by sex and age to determine the maximum matching possibility with the survey participants. This was then used to perform inferential statistics to identify important risk factors in each setting as well as for both settings combined. A mixed-effects logistic regression model was used to investigate associations between leptospirosis and potential risk factors, by calculating adjusted odds ratios (AOR) and allowing for random effects at the village level. Independent variables included in the analysis were age, sex, occupation, contact with animals, travel patterns and sharing of water sources used for consumption.

## Supplementary Information

Supplementary Information.
