# Supplementary material for: Human, animal, water source interactions and leptospirosis in Thailand
Source: Sci Rep. 2021 Feb 5;11:3215. doi: 10.1038/s41598-021-82290-5 (PMC7864926; doi:10.1038/s41598-021-82290-5)
Supplement: Supplementary file 1 — Supplementary Information. [file 41598_2021_82290_MOESM1_ESM.docx]

**Human, animal, water source interactions and leptospirosis in Thailand**

Udomsak Narkkul^1^, Janjira Thaipadungpanit^2,3^, Nattachai Srisawat^4^, James W. Rudge^5^, Metawee Thongdee^6^, Rungrawee Pawarana^7^, and Wirichada Pan-ngum^1,2*^

^1^ Department of Tropical Hygiene, Faculty of Tropical Medicine, Mahidol University, Bangkok, 10400, Thailand

^2^ Mahidol-Oxford Tropical Medicine Research Unit, Faculty of Tropical Medicine, Mahidol University, Bangkok, 10400, Thailand

^3^ Department of Clinical Tropical Medicine, Faculty of Tropical Medicine, Mahidol University, Bangkok, 10400, Thailand

^4^ Department of Medicine, Faculty of Medicine, Chulalongkorn University, Bangkok, 10400, Thailand

^5^ Communicable Diseases Policy Research Group (CDPRG), Department of Global Health and Development, London School of Hygiene & Tropical Medicine, London, United Kingdom

^6^ The Monitoring and Surveillance Center for Zoonotic Diseases in Wildlife and Exotic Animals (MoZWE), Faculty of Veterinary Science, Mahidol University, Nakhon Pathom 73170, Thailand

^7^ Center of Excellence for Biomedical and Public Health Informatics (BIOPHICS), Faculty of Tropical Medicine, Mahidol University, Bangkok, 10400, Thailand

Supplementary Information


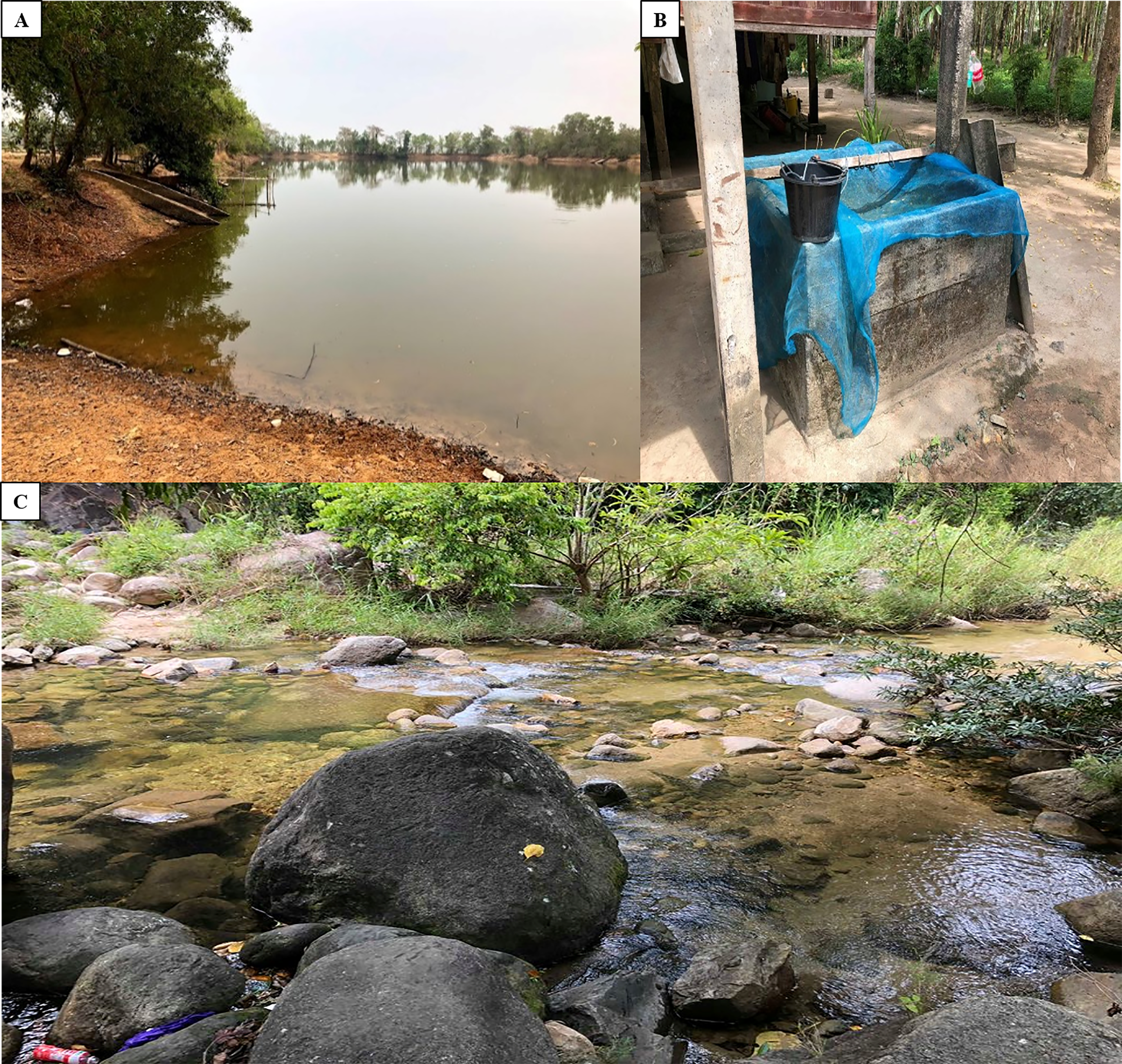


**Figure S1. A typical shared water source in the northeast of Thailand (A). A groundwater source owned by a household in the south of Thailand is usually close to or within the house. (B). Waterfalls are the only shared water sources in the south of Thailand (C).**

**Table S1. Numbers of humans and animals in each setting.**

| **Sisaket (SSK)** | | | **Nakhon Si Thammarat (NST)** | | |
| --- | --- | --- | --- | --- | --- |
| **Species** | **n** | **n (household)*** | **Species** | **n** | **n (household)*** |
| Human | 298 | 298 | Human | 299 | 299 |
| Chicken | 5343 | 235 | Chicken | 12667 | 161 |
| Cow | 136 | 37 | Cow | 136 | 36 |
| Buffalo | 408 | 83 | Buffalo | 0 | 0 |
| Pig | 73 | 12 | Pig | 285 | 22 |
| Dog | 195 | 126 | Dog | 334 | 193 |
| Cat | 186 | 67 | Cat | 266 | 135 |
| Duck | 419 | 35 | Duck | 185 | 11 |

**^*^** The household column shows the number of houses that had those animals.

**Questionnaire**

***Section 1: About the participant***

**Q1. Age** **□□** (in completed years).

**Q2. Sex** (circle one number)

O Male

O Female

**Q3. What is the highest level of education you have completed?**

O None

O Primary school

O High school

O University or higher

**Q4.** **What is your ethnicity?**

O Thai

O Cambodia

O Laos

O Other (specify)……………………………………….

**Q5. Geographical location** ________________ Province

________________ District

________________ Sub-district

**Q6.** **Location of residence** ________________ Latitude

________________ Longitude

**Q7. Occupation(s)**

**Primary/Main (MMM – MMM)**

O NONE

O agriculture

O farming

O fishing

O labourer (but not agriculture)

O student

O professional/office worker

O housewife

O shop worker

O others ……………………………

**Secondary/Spare-time (MMM – MMM)**

O NONE

O agriculture

O farming

O fishing

O labourer (but not agriculture)

O student

O professional/office worker

O housewife

O shop worker

O others ………………………

**Q8. Does your occupation(s) involve contact with animal?**

O Yes

O No (Go to Q10)

**Q9. If yes, please specify animal types (choose all that apply)**

O Rodent

O Cow or buffalo

O Farm animal including pig, duck, chicken

O Domestic pets

O Other (specify)……………………………………….

***Section 2: About the participant’s usual travel patterns***

**Q10. How often do you travel outside your Sub-district?**

O Most days of the week (i.e.,4-7 days per week)

O At least once a week but not most days (i.e.,1-3 times per week)

O At least once a month but not each week (i.e.,1-3 times per month)

O Less than once per month

O Never

**Q11. How often do you travel outside your district?**

O Most days of the week (i.e.,4-7 days per week)

O At least once a week but not most days (i.e.,1-3 times per week)

O At least once a month but not each week (i.e.,1-3 times per month)

O Less than once per month

O Never

**Q12. How often do you travel outside your province?**

O Most days of the week (i.e.,4-7 days per week)

O At least once a week but not most days (i.e.,1-3 times per week)

O At least once a month but not each week (i.e.,1-3 times per month)

O Less than once per month

O Never

**Q13. How often do you travel outside your country?**

O Most days of the week (i.e.,4-7 days per week)

O At least once a week but not most days (i.e.,1-3 times per week)

O At least once a month but not each week (i.e.,1-3 times per month)

O Less than once per month

O Never

***Section 3: Human-animal contacts***

**Q14. Do you have animal in your household?**

O Yes

O No (Go to Q23)

**Q15a. How many chicken does the household own?** **□□□□**

**Q15b. Where do they live?**

O Basement

O Around the house

O Sheikhs of the villages

O Other………………….

**Q15c. Which water source that animal used?**

O Same source with human

O Only animal

**Q16a. How many cow does the household own?** **□□□□**

**Q16b. Where do they live?**

O Basement

O Around the house

O Sheikhs of the villages

O Other………………….

**Q16c. Which water source that animal used?**

O Same source with human

O Only animal

**Q17a. How many buffalo does the household own?** **□□□□**

**Q17b. Where do they live?**

O Basement

O Around the house

O Sheikhs of the villages

O Other………………….

**Q17c. Which water source that animal used?**

O Same source with human

O Only animal

**Q18a. How many pig does the household own?** **□□□□**

**Q18b. Where do they live?**

O Basement

O Around the house

O Sheikhs of the villages

O Other………………….

**Q18c. Which water source that animal used?**

O Same source with human

O Only animal

**Q19a. How many dogs does the household own?** **□□□□**

**Q19b. Where do they live?**

O Basement

O Around the house

O Sheikhs of the villages

O Other………………….

**Q19c. Which water source that animal used?**

O Same source with human

O Only animal

**Q20a. How many cats does the household own**? **□□□□**

**Q20b. Where do they live?**

O Basement

O Around the house

O Sheikhs of the villages

O Other………………….

**Q20c.Which water source that animal used?**

O Same source with human

O Only animal

**Q21a. How many duck the household own**? **□□□□**

**Q21b. Where do they live?**

O Basement

O Around the house

O Sheikhs of the villages

O Other………………….

**Q21c.Which water source that animal used?**

O Same source with human

O Only animal

**Q22a. How many other…………... the household own**? **□□□□**

**Q22b. Where do they live?**

O Basement

O Around the house

O Sheikhs of the villages

O Other………………….

**Q22c.Which water source that animal used?**

O Same source with human

O Only animal

***Section 4: About the participant’s usual treatment seeking behaviour.***

**Q23. How many times did you feel sick or have fever since last month?**

………………. **times**

**Q24. What do you do when you feel sick or when you have fever?**

|  | Yes | No |
| --- | --- | --- |
| a) Pharmacy/shop | 1 | 2 |
| b) Health centre/clinic | 1 | 2 |
| c) Hospital | 1 | 2 |
| d) Traditional healer | 1 | 2 |
| e) Own treatment/remedy | 1 | 2 |
| f) Took no action | 1 | 2 |

**Q25. What do you suspect when you have fever or when you feel sick last time?**

**(Can choose more than one)**

O Influenza O Melioidosis

O Pesticide exposure O Hypertension or NCD

O Malaria

O Dengue

O Diarrhoea

O Other (specify)……………………………………….

**Q26a. Do you use any kind of protection to protect yourself from catching disease or spread disease?**

O Yes

O No

**Q26b. If yes, how do you use any kind of protection to protect yourself?**

O Wear boots or protective clothing

O Avoid to contact with animals that carry the disease

O Showering after down into the water that suspect contamination.

O Other (specify)……………………………………….

***Section 5: Water sources and animal habitats***

**FOR EACH WATER SOURCES**

**Q27. GIS location water sources** ________________ Latitude

________________ Longitude

**Q28. Does it have living animal around the water sources?**

O Yes

O No (Go to Q31)

**Q29. List of animal (More than 1)**

O Chicken

O Cow

O Buffalo

O Pig

O Dog

O Cat

O Duck

O Rodent

O Other (specify)……………………………………….

**Q30. How the water is used? (More than 1)**

O Drink

O Consume

O Agriculture

O Other (specify)……………………………………….

**Q31. What kind of water source that you use?**

O Close system (e.g. groundwater, pool, dam)

O Open system (e.g. river, irrigation)
